# The role of radiation therapy and systemic treatments in meningioma: The present and the future

**DOI:** 10.1002/cam4.6254

**Published:** 2023-06-27

**Authors:** Mario Caccese, Fabio Busato, Angela Guerriero, Marta Padovan, Giulia Cerretti, Marina Paola Gardiman, Vittorina Zagonel, Giuseppe Lombardi

**Affiliations:** ^1^ Department of Oncology, Oncology Unit 1 Veneto Institute of Oncology IOV‐IRCCS Padua Italy; ^2^ Department of Radiation Oncology Abano Terme Hospital Padua Italy; ^3^ General Pathology and Cytopathology Unit, Department of Medicine‐DMED University of Padua Padua Italy

**Keywords:** meningioma, new drugs, precision medicine, systemic treatment

## Abstract

Meningiomas are the most prevalent tumors of the central nervous system. Their standard treatment is surgery, which can be curative. Adjuvant radiotherapy treatment is reserved for newly diagnosed cases of grade II and grade III meningiomas in cases of recurrent disease or when surgery is not radical or feasible. However, around 20% of these patients cannot undergo further surgical and/or radiotherapy treatment. Systemic oncological therapy can find its place in this setting. Several tyrosine kinase inhibitors have been tested (gefitinib, erlotinib, sunitinib) with unsatisfactory or negative results. Bevacizumab has shown encouraging results in these settings of patients. Immunotherapy with immune checkpoint inhibitors has reported interesting results with modest objective response rates. Several ongoing studies are assessing different target therapies and multimodal therapies; the results are to be disclosed. Not only a better understanding of the molecular characteristics in meningiomas has allowed the gathering of more information regarding pathogenesis and prognosis, but in addition, the availability of new target therapy, immunotherapy, and biological drugs has widened the scope of potentially effective treatments in this patient population. The aim of this review was to explore the radiotherapy and systemic treatments of meningioma with an analysis of ongoing trials and future therapeutic perspectives.

## 1 INTRODUCTION

Meningioma is the most prevalent central nervous system (CNS) tumor, accounting for 39% of all CNS cancers^1^; the incidence rate is 9.12 per 100,000. The 5‐year survival rate is 88.2% for all grades.^1^ Several studies have demonstrated the genetic predisposition of patients with neurofibromatosis type 2 (NF2) to develop meningiomas: NF2 in an autosomal dominant disorder caused by alteration of the *NF2* gene with loss of function of the tumor suppressor protein,^2^ and 50%–75% of patients with this gene alteration have developed meningioma.^3^ These are typically grade II or III meningiomas with more aggressive behavior, a higher recurrence rate, and a poorer prognosis than those with sporadic onset.^4^, ^5^, ^6^, ^7^ The *NF2* gene alteration may also be a somatic mutation, since several somatic mutations have been identified in a variety of meningiomas including *AKT1*, *PIK3CA*, *SMO*, *TRAF7*, *POLR2A*, and *KLF4*.^7^, ^8^, ^9^, ^10^, ^11^, ^12^ On the other hand, a proportion of meningiomas contain germline mutations of *BAP1*, *SMARCB1*, and *SMARCE1* ^12^, ^13^, ^14^, ^15^; mutations affecting the *TERT* promoter or altered methylation patterns appear to be associated with more aggressive behavior.^16^, ^17^, ^18^ Meningiomas typically present as isointense in the T1‐weighted sequences and hyperintense in the T2/FLAIR‐weighted sequences, with marked contrast enhancement after gadolinium infusion on brain MRI. Nuclear medical images, such as those obtained with positron emission tomography (PET), are increasingly used for the examination of meningiomas, since the use of specific metabolic tracers can provide better information from both a quantitative and qualitative standpoint in specific settings.^19^ Considering that meningioma often expresses somatostatin receptor‐2, the use of tracers with somatostatin analogs (^68^Ga‐DOTATE or ^90^Y‐DOTATOC) is quite widespread, although to date it cannot be regarded as standard clinical practice. Several studies have demonstrated the ability of this type of examination to identify meningiomas even more effectively than brain MRI,^20^ and to obtain a better definition of the tumor extension for radiotherapy planning.^21^, ^22^, ^23^ Historically, the management of this type of tumor was limited to surgery or radiotherapy. Several preclinical data in the literature have evaluated new drugs, but the almost entirety of these treatments do not seem to determine objective benefits in the clinical setting. The reason for this could be due to the poor representation of cell culture and animal models that do not consider histological architecture, tumor microenvironment, and intratumor heterogeneity. All these parameters can significantly affect the activity of several drugs in the clinical setting.^24^ Furthermore, even the low rate of tumor growth and slow proliferation could be a limitation determining the failure in the clinical setting of treatments that had shown promising results in the pre‐clinical setting.^25^ As argued by some research groups, the implementation of new preclinical models, giving a more significant role to the molecular characteristics of the meningioma, could pave the way for new models that would be more transferable to the clinical setting.^25^ Another explanation could be, as discussed below, that, as mentioned below, clinical trials evaluating new treatments deriving from preclinical experiences are particularly underpowered with low numbers of patients treated. However, with the advent of new drugs and the increased knowledge of the molecular characteristics and therapeutic possibilities that can be derived from it, we have chosen to report the state‐of‐the‐art in the systemic treatment of meningioma. Considering the incidence of meningiomas, the aggressiveness of some forms (in particular for grade III) and the current surgical and radiotherapy techniques capable of prolonging the time to progression, which is often inevitable, it is likely that an increasing number of patients may prove in need of systemic treatment when locoregional treatments are not recommended anymore. For this reason, research must be able to guarantee increasingly personalized treatment, which can improve the outcome of these patients who, at the moment, have no therapeutic alternatives. Precision medicine and an accurate selection of patients from a molecular point of view, both in oncology in general and in neuro‐oncology, should be an absolute priority for the choice of treatments to be evaluated both in clinical trials and in clinical practice. The aim of this review was to explore the radiotherapy and systemic treatments of meningioma with an analysis of ongoing trials and future therapeutic perspectives. This study was approved by the Veneto Institute of Oncology IOV‐IRCCS ethics committee (No. 6/2023).

## 2 HISTOLOGY, GRADING, AND CLASSIFICATION

**FIGURE 1:**
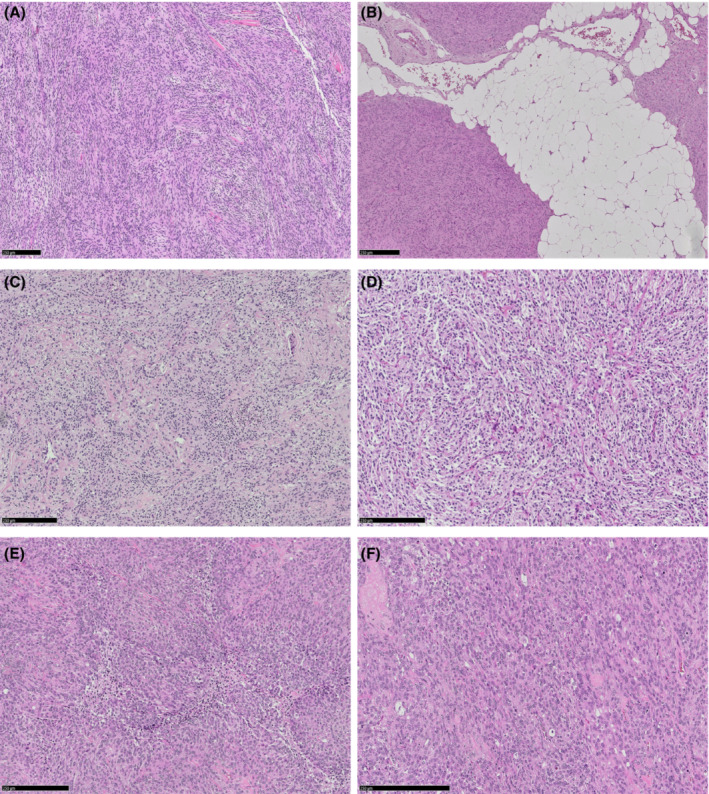
Histological features of meningiomas; grade 1 meningioma with fascicular pattern without features of atypia (A) and with adipose metaplasia (B); grade 2 meningioma with foci of spontaneous necrosis (C) cytological atypia and mitoses (D); anaplastic meningioma with necrosis, hypercellularity (E), marked cytological atypia and numerous mitoses (F).

A better understanding of the genomic structure of CNS tumors has permitted a reorganization of the classifications, not only on account of the morphological aspects, but also integrating the diagnosis with the molecular alterations. The histological features of meningioma specimens from patients treated at our institution are shown in Figure 1. This integrated diagnostics was first introduced by the 2016 WHO classification of CNS tumors.^26^ The 2016 WHO classification identified 15 different variants of meningiomas, divided into 9 WHO grade I subtypes, 3 WHO II (atypical), and 3 WHO grade III (anaplastic).^26^ The 2021 WHO classification^27^ retains the 15 distinct meningioma subtypes of the previous classification; however, the tumor grade is no longer defined by the meningioma subtype. The criteria of grade II and III meningiomas must be applied regardless of subtype^28^ instead. The real novelty of the most recent 2021 meningioma classification is that, although grading remains substantially based on histological criteria, molecular alterations play a fundamental role for diagnosis, classification, and grading. This aspect is paramount since improved classification and grading of these tumors enable a more accurate assessment of the risk of recurrence, a better prognostic analysis, and potentially the development of new and targeted treatments. The alteration of the tumor suppressor gene *NF2* is present in meningiomas arising in NF2 and in 60% of sporadic meningiomas.^28^ This is regarded as one of the earliest events in the carcinogenesis process, and meningiomas with *NF2* alterations tend to accumulate copy number variations, resulting in a subsequent increase in biological aggressiveness. Furthermore, numerous other recurrent mutations have been found, including *SMO*,^7^, ^9^ *AKT1*,^7^, ^9^ *TRAF7*,^7^, ^8^ *KFL4*,^7^, ^8^ *PIK3CA*,^10^ *BAP1*,^13^, ^14^ and *SMARCE1*,^29^, ^30^ which appear to be characteristic of certain subtypes and specific grading. The *TERT* promoter mutation has always been considered a parameter that characterizes meningiomas with a high risk of recurrence^16^, ^17^, ^31^, ^32^ in light of this new classification, this mutation, together with homozygous deletions of CDKN2A/B, is considered as an independent criterion for the diagnosis of grade III meningioma, irrespective of the histological criteria of anaplasia.^16^, ^27^, ^33^

## 3 RADIOTHERAPY

Establishing the optimal therapeutic option in the event of meningioma relapse is a multidisciplinary decision.^34^ Surgery is usually the first therapeutic option for first recurrences. In a recent article the overall TTR (time‐to‐retreatment) defined as the period between the initial surgery and consequent retreatment (surgery or RT) is 3.6 years; thereafter, progressively decreased to 0.4 years between the fifth and sixth surgeries.^35^ For this reason, surgery provides good local control in the case of a first recurrence. However, repeated surgery should be evaluated by a multidisciplinary board, as it is essential to assess the benefit‐to‐risk ratio for each patient. Various recurrence scenarios are conceivable in the event of recurrence.

### 3.1 Recurrence in the case of no previous RT

**FIGURE 2:**
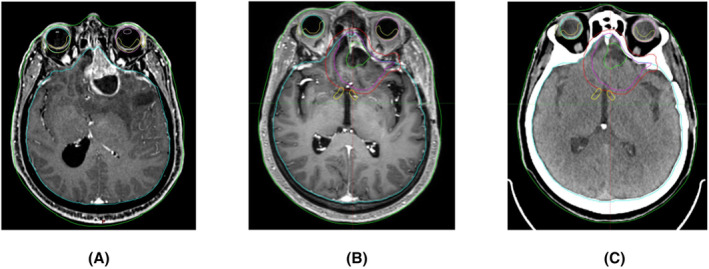
(A) Left frontal lesion radiologically suspected for high‐grade meningioma before surgery. MRI (B) and radiotherapy planning CT (C) after gross total resection: chiasma‐PRV in yellow, GTV in green, CTV in purple, and PTV in red.

Scientific evidence favors adjuvant RT for both grade II and grade III meningiomas.^36^ For this reason, nowadays meningioma recurrences with no previous irradiation are represented by grade I meningiomas. In most of the times grade I meningiomas are associated with optimal progression‐free survival (PFS) regardless of whether they are treated with gross total resection (GTR), external beam radiotherapy (EBRT), hypofractionated stereotactic radiotherapy (HFSRT), or radiosurgery. Huang et al.^37^ compared stereotactic radiotherapy (SRS; 2–16 Gy/1 fraction—100% isodose line to cover the target) to HFSRT (21Gy/3 fractions): no differences were found throughout the 5‐ to 10‐year local control rate (94.7% vs. 97.5% at 5 years; 74.4% vs. 91.4% at 10 years). EBRT (54 Gy in 2.0–1.8 Gy) provides optimal clinical control, comparable to GTR, SRS, or HFSRT. This technique is particularly recommended in proximity to organs at risk (OAR).^38^ A radiotherapy planned CT, pre‐surgery and post‐surgery brain MRI of meningioma patient treated at our institution are shown in Figure 2.

### 3.2 Recurrence in the case of previous RT

#### 3.2.1 Out‐of‐field recurrence

**FIGURE 3:**
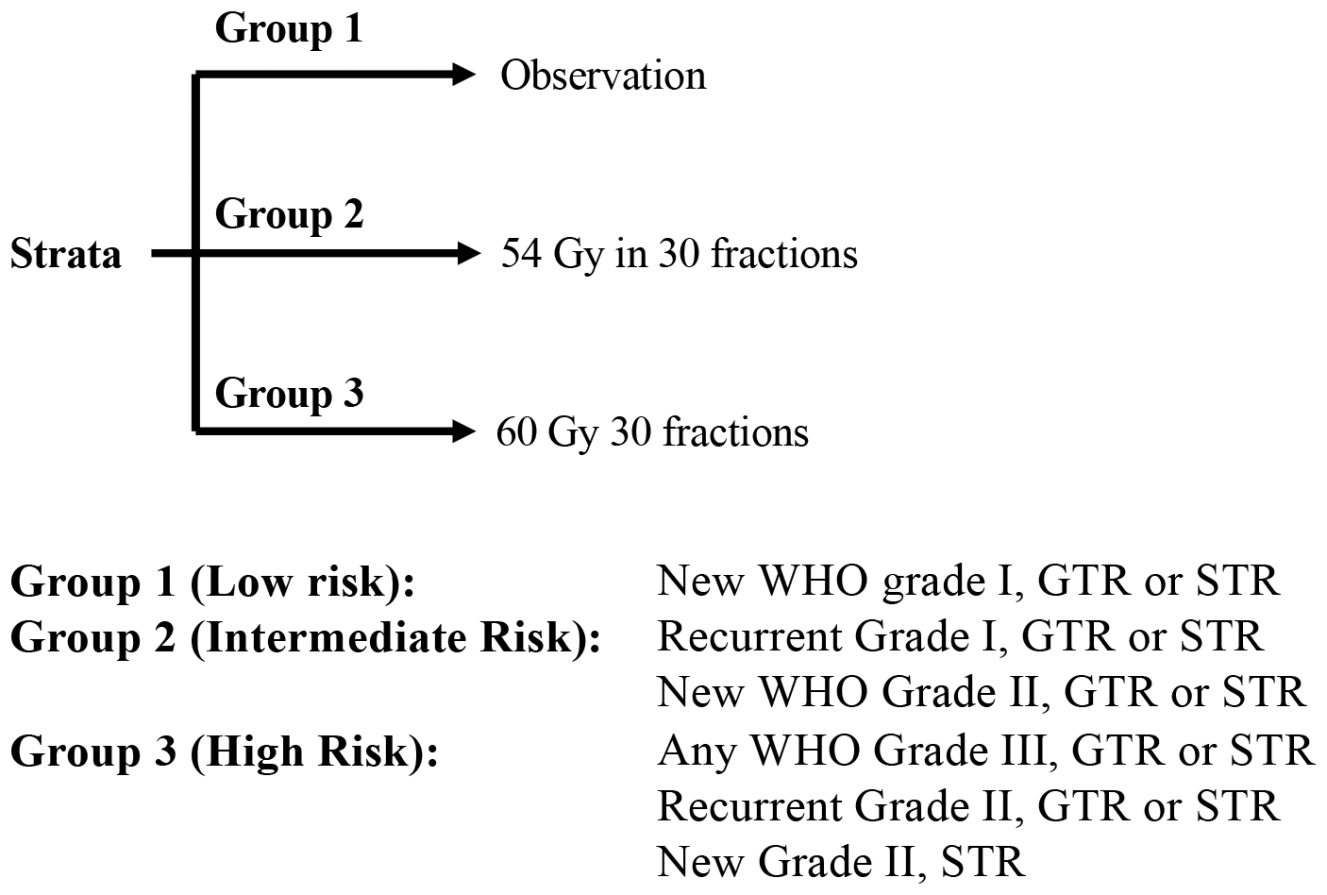
In the RTOG 0539 phase III clinical trial meningiomas were subdivided into three risk groups which were associated to different therapeutic approaches (observation, radiotherapy in 54 Gy, and radiotherapy in 60 Gy).

RTOG 0539 is an ongoing clinical trial investigating recurrent disease as well (Figure 3). It has established the importance of adjuvant RT as the standard of care for resected grade II and III meningiomas. The study divides meningiomas into three risk groups: Group 1 (newly diagnosed grade I meningioma), Group 2 (intermediate risk) with recurrent grade I meningiomas and totally resected grade II meningiomas, and Group 3 (high risk) with recurrent grade II, sub‐totally resected grade II, and grade III meningiomas. Group 2 was treated with a radiation dose of 54 Gy/30 fraction, while Group 3 was treated with a higher adjuvant RT dose (60 Gy/30 fraction). In Group 2, the 3‐year PFS was 93.8%—higher than the historical controls (70% after GTR alone and 90% following GTR + RT)^36^; in Group 3, 3‐year PFS was 58.8%.^39^ Whether adjuvant reduces tumor recurrence after gross total resection of grade II meningioma is still an open question. RT might in fact avoid future surgeries but this benefit is counterbalanced by possibility of chronic radiation induced side effects.^40^ Two ongoing clinical trials will compare PFS in newly diagnosed completely resected grade 2 meningiomas, treated with adjuvant RT or observation: the European ROAM/EORTC‐1308 trial (60 Gy/30 fractions) and the NRG BN 003 trial (59.4 Gy/33 fractions).

#### 3.2.2 In‐field recurrence

For in‐field recurrence, re‐irradiation (reRT) may be contemplated after a period of time, followed by a careful analysis of the risks and benefits associated with retreatment. In the case of re‐RT, tumor grade is the strongest predictor of PFS. Wojcieszynski et al.^41^ demonstrated that the 1‐year PFS is 17% for grade II–III meningiomas, and 92% for grade I meningiomas following re‐irradiation. This study highlights the efficacy of re‐irradiation for grade I recurrences and the limited efficacy of re‐irradiation for higher‐grade recurrences.^41^ Re‐RT can be delivered through SRS, HFSRT, EBRT, or heavy ion, with poor local control.

### 3.3 Radiosurgery

Re‐RT with radiosurgery should be carefully evaluated for high‐grade meningiomas, as it has demonstrated, with dose studied in this scenario of 14.2 Gy, poorer local control than with Grade 1 meningiomas.^42^ Another study shows that 1‐year PFS for patients with atypical or malignant tumors was 17%, compared to 90% for patients with grade 1, with dose of 10–12 Gy.^43^ These results are comparable to those obtained for first irradiation of high‐grade meningiomas.^44^

### 3.4 Hypofractionated stereotactic radiotherapy

HFSRT can be administered as a first treatment and at recurrence. In one study, the 24‐month local control rate was 81% in the primary HFSRT group, and 39% in the re‐irradiation group following HFSRT. Radionecrosis was present in 2% of the primary HFSRT group and in 20% of the re‐RT group after 24 months.^45^ Therefore, the benefits of re‐RT with HFSRT seem to be neglectable.

### 3.5 Brachytherapy

I‐125 implantation brachytherapy has been used to treat recurrent and previously irradiated high‐grade meningiomas: median time to progression was 20.9 months for Grade 2 and 11.4 months for Grade 3 meningiomas. The second course of RT was associated with complications for 17/42 patients: eight cases of radiation necrosis, six of wound breakdowns, four of hydrocephalus, three infections and two cases of pseudomeningocele. The oncological outcomes support brachytherapy as salvage treatment for recurrent meningiomas.^46^

### 3.6 Particle therapy

Particle therapy RT (protons and carbon ions) might be a good option in the case of OAR sparing and recent irradiation. Its efficacy, compared to conventional RT remains to be determined in future clinical trials.^47^, ^48^ Proton re‐RT has been shown to be safe; radionecrosis was detected in 13% of patients with half of the cases being grade 1 (asymptomatic) and the other half being symptomatic (grade 3).^49^ Re‐RT with protons has exhibited good local control rates.^49^ From a dosimetric point of view, protons show superior dose conformality and lower total integral dose when compared to photon treatment plans. Tumors like meningiomas, often close to critical OAR, might particularly benefit from protons dose conformality and health tissues sparing.^50^, ^51^ For instance, when considering proximity to the hippocampi, protons can reduce by 45% the dose to the hippocampi compared to IMRT and VMAT. Somehow, whether the dosimetric benefit converts to a clinical one in reducing late cognitive decline, as reasonably expected in second course irradiation, this remains to be demonstrated.^52^ Despite the lack of robust data, heavy‐ion re‐RT appears to be a viable therapeutic option, enabling high‐volume treatments and low toxicity rates.

## 4 SYSTEMIC TREATMENT

Systemic treatment for recurrent meningioma is typically reserved for cases in which surgery or radiotherapy treatment is no longer an option. Although there is currently no gold standard systemic treatment for recurrent/refractory meningioma, the use of different drugs have been investigated, with poor results in terms of survival and disease control. Another limitation is the scarcity of large and controlled clinical trials that can provide recommendations for these patients' treatments. In particular, the main limitations derive from the limited number of randomized prospective studies with good statistical power, from the low number of patients enrolled in clinical trials and the heterogeneous characteristics of the patients treated (grade I–II–III who which are known to lead to have different evolutions and responses to treatments). The main studies concerning systemic treatment in recurrent/refractory meningioma patients in recent years are summarized in Table 1.

**TABLE 1:** Main studies that have evaluated systemic treatment in recurrent/refractory meningioma patients.

Drug	Mechanism of action	Study	No. of patients	mPFS (months)	mOS (months)	6mPFS (%)	ORR (%)	DCR (%)
Hydroxyurea^62^	Ribonucleotide reductase inhibitor	Retrospective	60	2	7	10	0	35
Hydroxyurea^63^	Ribonucleotide reductase inhibitor	Retrospective	35	2	8	3	0	60
Hydroxyurea^64^	Ribonucleotide reductase inhibitor	Phase II	36	‐	‐	‐	5.5	41.5
Temozolomide^65^	Alkylating drug	Phase II	16	‐	7	0	‐	‐
Irinotecan^66^	Topoisomerase I inhibitor	Phase II	16	‐	7	6	0	81
Trabectidin^73^	DNA intercalator	Randomized Phase II	61	2.4	11.3	21.1	1.8	38.6
Octreotide^76^	Somatostatin analogs	Phase II	16		7.5	44	31	31
Pasireotide^78^	Somatostatin analogs	Phase II	18 (grade II–III)	15 (weeks)		17		67
Gefitinib/Erlotinib^82^	EGFR inhibitor	Phase II	25	10 (weeks)	23	‐25 (grade I) ‐29 (grade II–III)	0	32
Imatinib^83^	Multikinase inhibitor	Phase II	22	‐3 (grade I) ‐2 (grade II–III)		‐45 (grade I) ‐0 (grade II–III)	0	47
Sunitinib^84^	Multikinase inhibitor	Phase II	36	5.2	24.6	42	5.7	77.1
Vatalanib^85^	Multikinase inhibitor	Phase II	25	‐7.6 (grade II) ‐3.6 (grade III)	‐26 (grade II) ‐23 (grade III)	‐64.3 (grade II) ‐37.5 (grade III)	0	68.2
GSK2256098^86^	FAK inhibitor	Phase II	36	‐	‐	‐83 (grade I) ‐33 (grade II–III)	2.7	69.4
Evorolimus + octreotide^88^	mTOR inhibitor + Somatostatin analogs	Phase II	20	6.6	‐	55	0	‐
Everolimus + Bevacizumab^89^	mTOR inhibitor + VEGF Monoclonal antibody	Phase II	17	22	23.8	69	0	88
Bevacizumab^79^	VEGF binding monoclonal antibody	Retrospective	14	17.9	NR	85.7	7	86
Bevacizumab^80^	VEGF binding monoclonal antibody	Retrospective	15	26 (weeks)	15	43.8	0	100
Bevcizumab^81^	VEGF binding monoclonal antibody	Phase II	42	22	35	87	2.4	88.4
Pembrolizumab^87^	Anti‐PD1 monoclonal antibody	Phase II	26	7.6	20.2	48	0	69.2
Nivolumab^89^	Anti‐PD1 monoclonal antibody	Phase II	25	5.5	30.9	42.4	4	64
Interferon‐alfa^88^		Retrospective	35	12 (weeks)	5	17	0	63

Abbreviations: DCR, disease control rate; ORR, objective response rate.

### 4.1 Chemotherapy

Several chemotherapy drugs have been evaluated as treatments for recurrent/refractory meningioma, with disappointing results in terms of activity and efficacy. Hydroxyurea remains the most researched chemotherapy to date; despite initially encouraging preclinical results,^53^ its efficacy was not confirmed in clinical trials. Chamberlain and Johnston^54^ investigated hydroxyurea treatment in 60 patients with recurrent/refractory grade 1 meningioma. No radiological response was observed in the framework of a disease control rate (DCR) of 35% and a median progression‐free survival (mPFS) of only 2 months. The prospective data on the use of hydroxyurea comes from a phase II study^55^ in which 43 patients with grade I and II meningioma were treated. The objective response rate was low (5%) with a DCR of 36%. Temozolomide, an alkylating chemotherapy drug, was also tested in a phase II prospective study,^56^ with modest results; all patients progressed after the first 2‐cycles, with a median overall survival of 7 months (95% CI: 7–8 months). Irinotecan, a chemotherapy drug in the camptothecin family, was also tested in a phase II study^57^ in patients with recurrent grade I meningioma, after surgery and radiotherapy had failed. No patient displayed a neuroradiographic partial (PR) or complete response (CR) The median OS of 7 months, and a median time to progression of 5 months. Given the presence of progesterone receptors in the meningioma tissue,^58^ mifepristone, an oral progesterone antagonist, has been tested in several clinical studies as a possible rescue therapy for patients with recurrent meningioma; six clinical trials demonstrated a low rate of objective responses and symptom improvement in inoperable meningioma.^59^ Trabectedin is an antineoplastic agent that was formerly extracted from the Caribbean marine tunicate *Ecteinascidia turbinata* and is now entirely synthesized. Its mechanism of action involves binding with the DNA's minor groove and subsequent formation of trabectedin‐DNA adducts, resulting in the interruption the cell cycle.^60^ Despite encouraging data derived from trabectedin activity in grade II and III meningioma cell lines in previous preclinical studies,^61^ a recently published phase II clinical trial did not confirm these results: 90 patients with recurrent grade II or III meningiomas were randomly assigned 2:1 to receive trabectedin or local standard of care (LOC). Trabectedin did not improve PFS and OS, and its toxicity profile was worse than that of LOC.^62^ Several studies have demonstrated that a large percentage of meningiomas (close to 90%) express receptors for somatostatin.^63^, ^64^ For this reason, several studies have explored the possibility of using somatostatin analogs as a potential treatment for recurrent meningiomas. In a small prospective trial,^65^ 16 meningioma patients with confirmed somatostatin receptor expression via long‐acting somatostatin agonist SPECT scanning were treated with octreotide. The objective response rate (ORR) was 31%, while the 6‐month PFS was 44% and the mOS was 7.5 months (range 3–20). Another phase II study^66^ evaluated the use of a different long‐acting somatostatin analogs (Pasireotide‐LAR) in patients with recurrent grade II and III meningiomas. This study did not support the previous trials' findings of a 6‐month PFS (17%) and a mPFS of 15 weeks (95% CI: 8–20).

### 4.2 Target therapy

It is common knowledge that certain growth factors, such as epidermal growth factor (EGF), platelet‐derived growth factor (PDGF), and vascular endothelial growth factor (VEGF) are overexpressed in meningiomas, although their role in the pathogenesis of meningioma remains unclear.^67^ Several studies have explored the possibility of using tyrosine kinase inhibitors as a treatment for recurrent meningioma. In a single‐arm phase II study^68^ two EGFR inhibitors were tested in patients who had been administered more than two prior lines of therapy. A total of 25 patients were enrolled and treated, 8 (32%) with grade I meningioma, 9 (36%) with atypical meningioma, and 8 (32%) with anaplastic meningioma. Sixteen (64%) patients were administered gefitinib with a daily 500 mg dosage, while nine (36%) received erlotinib (150 mg/day). The treatment was well tolerated but did not prove to be active in this patient setting; treatment did not improve survival (PFS and OS); no objective response was reported, with disease stability as the best response in 32% of patients. Most meningiomas appear to express the AA and BB ligands of PDGF and the PDGF‐beta receptor, demonstrating, in vitro, how these can play a role in supporting the proliferation of meningioma cells.^69^, ^70^ For this reason, a phase II study^71^ has evaluated the use of imatinib, a PDGFR inhibitor, as a treatment for heavily pretreated recurrent grade I–III meningiomas. Twenty‐three patients were enrolled in this study (13 of them with grade I, 5 with grade II, and 5 with grade III meningioma), but only 22 patients were treated with imatinib at a dosage of 600 mg/day for the first 4 weeks (cycle 1). The dosage was then gradually increased up to 800 mg/day for subsequent cycles. The treatment was well tolerated but failed to show activity in this subset of patients. No radiological response was observed: 13 patients progressed at first scan and 9 had stable disease (SD) as best response. Sunitinib, a multi‐kinase inhibitor capable of targeting both the VEGF receptor (VEGFR) and the PDGF receptor (PDGFR), has been studied as a treatment in recurrent meningioma. In a prospective, open‐label, single‐arm phase II study,^72^ sunitinib was tested in recurrent and pretreated grade II–III meningioma patients. A total of 36 patients were enrolled: 6 with grade III meningioma and 30 with grade II meningioma. The 6mPFS was 42%, and the mOS was 24.6 months (95% CI: 16.5–38.4 months). The study reached its primary endpoint (6mPFS); however, the authors concluded that a randomized study with greater statistical power would be useful to confirm these data. Vatalanib, an oral VEGFR/PDGFR inhibitor, was tested in a phase II clinical trial as a treatment for recurrent grade I–III meningioma patients.^73^ A total of 25 patients were treated: 8 patients with grade III meningioma, 14 patients with grade II, and 2 patients with grade I meningioma; 1 patient had a hemangiopericytoma. Approximately two‐thirds (68.2%) of evaluable patients showed SD as the best response. For grade II patients, the 6mPFS was 64.3% and the mOS was 26 months. In contrast, the 6mPFS for grade III patients was 37.5% and a mOS of 23 months. The phase II Alliance/NCI A071401 study, in which meningioma patients were treated with different target therapies based on the type of mutation found in the tumor tissue (SMO inhibitors, AKT inhibitors, CDK inhibitors, and FAK inhibitors) is still ongoing, but enrollment for the cohort with FAK inhibitors has been completed,^74^ with the recruiting and treatment of 36 patients. The 6mPFS for patients with grade I meningioma was 83% (95% CI: 52–98), as opposed to 33% (95% CI: 16–55) in those with grade II–III meningioma, with 24 patients achieving SD as the best response to treatment and with only one partial response. In meningiomas, disruptions of the mTOR pathway, and the possible reduced tumor proliferation in vitro due to the inhibition of said pathway, is common knowledge.^75^ For this reason, everolimus, a mTOR kinase inhibitor, has been tested in meningioma patients in combination with octreotide or antiangiogenic drugs such as bevacizumab. Specifically, the CEVOREM phase II study^76^ tested the combination of everolimus and octreotide in patients with recurrent meningioma who were not candidates for additional surgical and/or radiotherapy treatments. This study enrolled 20 patients (2 grade I, 10 grade II, and 8 grade III meningiomas) who were treated with everolimus at a dosage of 10 mg/day on days 1–28 and octreotide at 30 mg/day on day 1. The 6mPFS was 55% (95% CI: 31.3–73.5), while OS at 6 and 12 months was 90% (95% CI: 65.6–97.4) and 75% (95% CI: 50.0–88.7). Everolimus was also evaluated in combination with bevacizumab in a prospective phase II study^77^ that treated 17 patients with meningioma recurrence (5 with grade I, 7 with grade II, 4 with grade III, and 1 meningioma with an unknown grading). The median progression‐free survival (mPFS) was 22 months (95% CI: 4.5–26.8); 15 patients (88%) had SD as the best response and 6 achieved SD >12 months. While the PFS data from this study are encouraging, they do not appear to be different from the retrospective studies using bevacizumab, discussed below.

### 4.3 Bevacizumab

The VEGF pathway has been extensively studied in meningiomas.^78^ Among the antiangiogenic drugs available in the oncological therapeutic armamentarium, bevacizumab is the most studied as a treatment for meningiomas, even though in retrospective trials that enrolled a small number of patients from a highly heterogeneous population. One of the first retrospective studies on the use of bevacizumab was published in 2012 by Lou et al.^79^ who assessed the efficacy of this type of treatment in 14 heavily pretreated patients with grade I–III meningioma: 6mPFS of 86%, regardless of meningioma grade and whether bevacizumab was administered alone or in combination with chemotherapy. Another retrospective study evaluated bevacizumab in pretreated atypical (II) or anaplastic (III) meningioma patients.^80^ Fifteen patients were treated: the best radiological response was SD, with a 6mPFS of 43.8% (95% CI: 15.7–69.1). One of the few phase II studies evaluating bevacizumab in 50 patients with meningioma was recently published by Kumthekar et al.^81^ The study involved 10 patients with grade I, 20 with grade II, and 12 with grade III meningiomas. The 6mPFS was 87%, 77%, and 46% for grade I, II, III meningiomas respectively. The median overall survival (OS) was 35, 41, and 12 months for grades I, II, and III, respectively. SD was the best response in the majority of the patients. Treated patients showed good tolerance to the drug, reporting hypertension (42.2%), proteinuria (35.6%), and fatigue (31.1%) as the most common adverse events.

### 4.4 Immunotherapy

Immunotherapy, in particular with immune checkpoint inhibitors, has revolutionized the paradigm of systemic treatment in oncology, showcasing excellent results in different types of cancers. Unfortunately, this advantage has not yet been demonstrated in the neurooncological field. The study of this aspect, and for as concerns meningiomas as well, is generating significant interest^82^ for a better understanding of the immunological and genomic characteristics of the tumor immune cells infiltrating can provide applications for the development of potentially more personalized treatments. Interferon‐α (IFN‐α) belongs to a family of proteins which are physiologically produced by the cells of the immune system; in addition to antiviral, antiproliferative, and immunomodulatory activity, it is able to activate some intracellular pathways that impact cell growth and division, as well as to modulate various activities of the immune system.^83^ Following confirmation of the growth inhibition of meningioma cells in vitro,^84^ a prospective study^85^ treated 35 grade I meningioma patients with interferon‐α, resulting in a median time to progression of 7‐month, even though none of the patients have achieved partial or complete response to treatment. The expression of PD‐L1 (programmed death‐ligand 1) used as possible biomarkers of response to immunotherapy with immune checkpoint inhibitors as found to be present in a variable percentage of meningiomas (5%–80%), with levels of expression directly proportional to histological grading.^86^ Immune checkpoint inhibitors have been tested in meningioma patients: a phase II study^87^ used pembrolizumab (an anti‐PD‐1 immune checkpoint inhibitor) in 26 patients with grade II and III meningiomas and achieved a 6mPFS of 48% (90% CI: 31–66) and mOS of 20.2 months (90% CI:14.8–25.8). No patients have achieved partial or complete response, while 18/26 patients had stable disease as the best response. Another phase II study^88^ has evaluated the use of another anti‐PD‐1 immune checkpoint inhibitor (Nivolumab) in patients with grade II and III meningiomas. Said study failed to assess the 6mPFS, which was 42.4% (95% CI: 22.8–60.7) with a mOS of 30.9 months (95% CI: 17.6–NA). Only one patient achieved partial response as the best response. Although the main objective of the study was not achieved, one patient benefited from this type of treatment, and this requires validation to improve patient selection for this therapeutic approach. Several trials are currently evaluating the use of immune checkpoint inhibitors in meningioma patients. One of these studies are evaluating the combination of nivolumab plus ipilimumab with radiotherapy treatment for patients with grade II–III meningiomas (NCT02648997, NCT03604978). Other immune checkpoint inhibitors are also being tested in a number of studies (NCT03267836—avelumab; NCT03279692, NCT03016091, and NCT04659811—pembrolizumab).

## 5 FUTURE PERSPECTIVES AND ONGOING TRIALS

In the future, further efforts will be essential to identify systemic oncological treatments that provide patients with an effective therapeutic chance when locoregional treatments are no longer recommended. Currently, several trials in different pre‐clinical phases are evaluating the possibility of using new drugs that have never before been tested in a clinical setting in patients with meningioma. Alpelisib, a PI3Kα inhibitor already approved for the treatment of breast cancer, is currently being evaluated for meningioma patients in a phase I study (NCT03631953), together with trametinib a MEK inhibitor. Currently there is no data available. Apatinib, an oral VEGFR2 tyrosine kinase inhibitor, is being evaluated in a prospective single‐arm study (NCT04501705) in patients with recurrent grade II and III meningiomas. Brigatinib, a tyrosine kinase inhibitor that targets *ALK*, *ROS1*, and *IGF‐1R*, is currently being investigated in a phase II basket trial (NCT04374305) enrolling patients who have been diagnosed with NF2, including meningiomas. CDK4/6 cyclin inhibitors are being evaluated in a phase II study (NCT03220646), which used abemaciclib, extended to different types of brain tumors; the primary endpoint is the activity expressed in the radiographic response rate. Other studies are now investigating the efficacy, activity, and safety of various drugs in combination with radiotherapy, notably panobinostat (a histone deacetylase inhibitor) combined to stereotaxic radiotherapy (NCT01324635—the study has been completed but its results are not available yet); nivolumab and ipilimumab (anti‐PD1 and anti‐CTLA4, respectively) in combination with radiotherapy (NCT03604978); pembrolizumab (anti‐PD1) plus stereotaxic radiotherapy (NCT04659811); avelumab (anti‐PD‐L1) in a neoadjuvant setting together with proton radiotherapy followed by surgery (NCT03267836). Nuclear medicine could also lead to new therapeutic strategies like receptor radionuclide therapy. Somatostatin analogs are coupled by a β‐emitter, usually 90Yttrium (Y), in order to target somatostatin receptors on meningioma cells. A recent meta‐analysis of refractory meningioma^89^ has shown that, despite all studies including small cohorts, the treatment is well tolerated and disease control is achieved in 63% of patients, thus demanding for more robust results.^90^ Due to the nature of the data on systemic therapies currently available for recurrent meningiomas, it is certainly still necessary to develop effective drugs based on solid scientific evidence that can guarantee a better outcome for patients. A deeper understanding of the genetic, epigenetic, and immunological alterations in this type of cancer could certainly pave the way for new therapeutic perspectives. The strengths of this review are the detailed and particularly up‐to‐date descriptions of the radiotherapy indications and techniques, and of the possible systemic oncological therapies currently available (or under study) for the treatment of meningiomas. The emphasis on future research perspectives which may be able to address the current unmet needs for the treatment of these patients.

## AUTHOR CONTRIBUTIONS

**Mario Caccese:** Conceptualization (equal); data curation (equal); funding acquisition (equal); investigation (equal); methodology (equal); resources (equal); software (equal); supervision (equal); validation (equal); visualization (equal); writing – original draft (equal); writing – review and editing (equal). **Fabio Busato:** Conceptualization (equal); data curation (equal); formal analysis (equal); investigation (equal); methodology (equal); resources (equal); software (equal); supervision (equal); validation (equal); visualization (equal); writing – original draft (equal); writing – review and editing (equal). **Angela Guerriero:** Data curation (equal); formal analysis (equal); investigation (equal); methodology (equal); resources (equal); software (equal); supervision (equal); validation (equal); visualization (equal); writing – review and editing (equal). **Marta Padovan:** Data curation (equal); formal analysis (equal); investigation (equal); methodology (equal); resources (equal); software (equal); supervision (equal); validation (equal); visualization (equal); writing – review and editing (equal). **Giulia Cerretti:** Data curation (equal); formal analysis (equal); investigation (equal); methodology (equal); resources (equal); software (equal); supervision (equal); validation (equal); visualization (equal); writing – review and editing (equal). **Marina Paola Gardiman:** Data curation (equal); formal analysis (equal); investigation (equal); methodology (equal); resources (equal); software (equal); supervision (equal); validation (equal); visualization (equal); writing – review and editing (equal). **Vittorina Zagonel:** Conceptualization (equal); data curation (equal); formal analysis (equal); funding acquisition (equal); investigation (equal); methodology (equal); resources (equal); software (equal); supervision (equal); validation (equal); visualization (equal); writing – original draft (equal); writing – review and editing (equal). **Giuseppe Lombardi:** Conceptualization (equal); data curation (equal); formal analysis (equal); funding acquisition (equal); investigation (equal); methodology (equal); resources (equal); software (equal); supervision (equal); validation (equal); visualization (equal); writing – original draft (equal); writing – review and editing (equal).

## FUNDING INFORMATION

This study was supported by “Ricerca Corrente 2022 by the Italian Ministry of Health.”

## CONFLICT OF INTEREST STATEMENT

All authors declare no conflicts of interest.

## INFORMED CONSENT STATEMENT

Informed consent was obtained from patients for use/publication of the images.

## Data Availability

Data available on request due to privacy/ethical restrictions
